# Natural Fungal Endophytes From *Noccaea caerulescens* Mediate Neutral to Positive Effects on Plant Biomass, Mineral Nutrition and Zn Phytoextraction

**DOI:** 10.3389/fmicb.2021.689367

**Published:** 2021-07-06

**Authors:** Loïc Yung, Catherine Sirguey, Antonin Azou-Barré, Damien Blaudez

**Affiliations:** ^1^Université de Lorraine, CNRS, LIEC, Nancy, France; ^2^Université de Lorraine, INRAE, LSE, Nancy, France

**Keywords:** hyperaccumulator, inoculation, plant-fungus interaction, plant growth promotion, trace elements

## Abstract

Phytoextraction using hyperaccumulating plants is a method for the remediation of soils contaminated with trace elements (TEs). As a strategy for improvement, the concept of fungal-assisted phytoextraction has emerged in the last decade. However, the role played by fungal endophytes of hyperaccumulating plants in phytoextraction is poorly studied. Here, fungal endophytes isolated from calamine or non-metalliferous populations of the Cd/Zn hyperaccumulator *Noccaea caerulescens* were tested for their growth promotion abilities affecting the host plant. Plants were inoculated with seven different isolates and grown for 2 months in trace element (TE)-contaminated soil. The outcomes of the interactions between *N. caerulescens* and its native strains ranged from neutral to beneficial. Among the strains, *Alternaria thlaspis* and *Metapochonia rubescens*, respectively, isolated from the roots of a non-metallicolous and a calamine population of *N. caerulescens*, respectively, exhibited the most promising abilities to enhance the Zn phytoextraction potential of *N. caerulescens* related to a significant increase of the plant biomass. These strains significantly increased the root elemental composition, particularly in the case of K, P, and S, suggesting an improvement of the plant nutrition. Results obtained in this study provide new insights into the relevance of microbial-assisted phytoextraction approaches in the case of hyperaccumulating plants.

## Introduction

Among the different phytoremediation approaches applied, phytoextraction using hyperaccumulating plants is a method for the remediation of soils contaminated with trace elements (TE) (Robinson et al., 1998; Zhao et al., 2003; McGrath et al., 2006). Among the TE targeted for phytoextraction applications, cadmium (Cd) and zinc (Zn) are of particular interest, as they are found at elevated levels in soils mainly due to anthropic activities such as mining, smelting, the use of domestic and industrial wastes or the burning of fossil fuels, causing serious environmental problems worldwide (Alloway, 2013; Su et al., 2014). Cadmium is considered one of the most toxic non-essential elements for plants (Clemens and Ma, 2016), and Zn has phytotoxic effects when present in excess in plants (Kabata-Pendias, 2010). Among the various hyperaccumulators, *Noccaea caerulescens* (J. & C. Presl) F. K. Mey (Brassicaceae) is one of the species with the highest ability to tolerate and accumulate Cd and Zn (Lombi et al., 2000; McGrath et al., 2006). *In situ*, *N. caerulescens* can concentrate up to 2,890 μg Cd g^–1^ and 53,450 μg Zn g^–1^ in dry shoots (Reeves et al., 2001); however, significant variations in metal accumulation depend on the populations and edaphic type (Gonneau et al., 2014; Sterckeman et al., 2017). Among all the known populations, the calamine Ganges ecotype is the most studied due to its high capacity to accumulate Cd and its tolerance to Cd and Zn (Assunção et al., 2003; Gonneau et al., 2014).

Recent studies aimed to optimize the phytoextraction efficiency of *N. caerulescens* by maximizing both the biomass production and metal concentration in plants. According to Escarré et al. (2013), shoot concentrations tend to be restricted to a physiological maximum, limiting the possibility of improving this factor. There is, however, high potential for improvement in the biomass factor in the case of *N. caerulescens* (Sterckeman et al., 2017). Various levers have been investigated to increase biomass production, such as the selection of high-growth rate cultivars (Sterckeman et al., 2017, 2019) and the development of adequate cultural practices (Maxted et al., 2007; Jacobs et al., 2018a,b; Rees et al., 2020), including the addition of biological amendments. Indeed, we previously demonstrated that several DSE strains isolated from poplar roots collected from TE-contaminated sites were able to colonize the roots of *N. caerulescens*, with plant responses ranging from neutral to beneficial (Yung et al., 2021b). We also identified two promising fungal strains for the fungal-assisted phytoextraction of Cd and Zn with *N. caerulescens*. However, the diversity of the fungal endophytome of Zn- and Cd-adapted *N. caerulescens* and the effect of potentially plant-growth promoting (PGP) native fungal strains on the phytoextraction potential of the plant have not yet been investigated.

Over the last decade, the concept of microbial-assisted phytoremediation (MAP) has developed. When applied to phytoextraction, it consists of inoculating metal-(hyper)accumulating plants with plant-associated microorganisms to enhance metal recovery rates (Thijs et al., 2016; Benizri et al., 2021). MAP is based on the ability of PGP microorganisms such as PGP rhizobacteria (Hansda et al., 2014), endophytic bacteria (Ma et al., 2016), arbuscular mycorrhizal fungi (Ciadamidaro et al., 2017; Miransari, 2017; Berthelot et al., 2018), ectomycorrhizal fungi (Ciadamidaro et al., 2017; Phanthavongsa et al., 2017; Gil-Martínez et al., 2018), and endophytic fungi (Lacercat-Didier et al., 2016; Berthelot et al., 2017, 2018; Deng and Cao, 2017) to enhance plant development, health, tolerance to abiotic stress and resistance to phytopathogens (Kong and Glick, 2017; Thijs et al., 2016; Otero-Blanca et al., 2018; Durand et al., 2021). Additionally, root-associated microorganisms are able to increase the mobility of TEs in the soil, suggesting that their presence is essential for plants to reach their full phytoextraction potential (Lebeau et al., 2008; Sessitsch et al., 2013). In this context, hyperaccumulating plants and associated microorganisms could be considered together as plant-microbiome superorganisms (Bosch and McFall-Ngai, 2011), in which the microbial component acts as a dynamic system, increasing the adaptability of the plant-microbiome superorganism under environmental pressure (Durand et al., 2021). Indeed, in the case of TE-enriched soils, the host plant can promote metal-tolerant beneficial microorganisms from the enormous and diverse pool of microorganisms present in the bulk soil (Becerra-Castro et al., 2012; Álvarez-López et al., 2016; Yung et al., 2021a). Detecting and isolating these preferentially selected microorganisms represents a preliminary step of MAP.

The diversity of potentially PGP fungi and bacteria associated with *N. caerulescens* has been poorly investigated (Visioli et al., 2015a). Bacterial endophytes of *N. caerulescens* were mainly studied utilizing cultivation-dependent methods (Aboudrar et al., 2007; Visioli et al., 2014). Recently, 16S DNA profiling has been used to investigate root-associated bacterial communities (Visioli et al., 2019) and endophytic bacterial communities associated with seeds (Durand et al., 2021) of *N. caerulescens.* Several promising strains of metal-resistant or PGP endophytic bacteria have already been isolated (Visioli et al., 2014; Fones et al., 2016; Burges et al., 2017), and their potential to improve biomass production and metal accumulation has been tested *in planta* (Visioli et al., 2015b; Burges et al., 2017). Like most plants belonging to the Brassicaceae family, *N. caerulescens* is a recognized non-mycorrhizal plant, suggesting that endophytic fungi may be the dominant group of symbiotic fungi inhabiting its roots. However, its fungal endophytome has been poorly studied thus far. The first data about the cultivable fungal diversity associated with the roots and leaves of *N. caerulescens* grown on Ni-enriched soils were recently reported (Ważny et al., 2021). Among the 13 taxa isolated, most of the strains showed a positive effect on the production of plant biomass and on Ni accumulation in roots and/or shoots. Additionally, Ważny et al. (2021) reported that shoots accumulated more Ni when the plants were inoculated with a strain of *Phomopsis columnaris* isolated from *N. caerulescens*, highlighting the importance of selecting indigenous strains. However, indigenous fungal strains have not been tested for their effect on Cd/Zn phytoextraction.

By examining fungal endophytes isolated from calamine or non-metalliferous populations of *N. caerulescens*, this study therefore aimed to (i) characterize some of their PGP abilities and their influence on the mobility of TE through *in vitro* tests, (ii) assess their capacity to colonize the roots of the hyperaccumulating plant *N. caerulescens*, (iii) study their effect on plant growth, leaf pigment contents and mineral nutrient status, and (iv) determine their impact on the accumulation and phytoextraction of Cd and Zn.

## Materials and Methods

### Fungal Endophyte Isolation and Identification

Isolation of fungal endophytes from the roots and leaves of 58 individuals of *N. caerulescens* from seven populations (around eight plants per population) collected in two regions of France (Grand Est and Occitanie) was performed, with plants from each region corresponding to a genetic subunit (Gonneau et al., 2017). The full description of the cultivable fungal endophytome of *N. caerulescens* will be described elsewhere (unpublished results). Once collected, plant parts were carefully washed with tap water, surface-sterilized by immersion in 30% (v/v) H_2_O_2_ for 30 s and finally rinsed three times with sterile distilled water. For each plant, 15 root segments of 1 cm and 15 leaf fragments of 1 cm^2^ were placed on malt extract agar (MEA, malt extract: 12 g/L and agar: 15 g/L, pH 5.5) at 24°C in the dark. Ampicillin and chloramphenicol (100 μg/ml) were also added to the medium to avoid bacterial development. After 15 days of growth, fungal mycelium growing out of the plant tissues was isolated and subcultured in new MEA plates for 3 weeks.

For this experiment, we considered seven strains that were molecularly identified. Fungal DNA was first extracted (100 μL) using a REDextract-N-AmpTM Plant PCR kit (Sigma-Aldrich, Saint-Quentin Fallavier, France) according to the manufacturer’s instructions. The internal transcribed spacer (ITS) region was then amplified using ITS1 and ITS4 primers (White et al., 1990). Twenty microliters of a mixture containing 0.1 μM of each primer, 1 μL of the fungal extract, 8.2 μl of water and 10 μl of REDExtract-N-Amp PCR ready mix was used. The following PCR program was used: 3 min denaturation at 94°C, followed by 40 cycles of 94°C for 30 s, 55°C for 30 s and 72°C for 75 s and 10 min final extension at 72°C. The DNA quality and quantity were assessed by agarose gel electrophoresis using Molecular Imager^®^ Gel Doc^TM^ XR (Bio-Rad, United States). Fungal amplicons were then sequenced using the Sanger method (Eurofins, Germany). The obtained ITS sequences were used to retrieve similar sequences from GenBank using the NCBI BLASTn program.

### Indole Acetic Acid Production

The quantities of indole acetic acid (IAA) produced by the seven strains were determined by inoculating 40 mL of potato dextrose broth (PDB, pH 5.2) supplemented or not with 0.2% (m/v) tryptophan with six discs of mycelium of 5 mm in diameter (*n* = 3). The fungal cultures were incubated at 24°C for 10 days with constant shaking at 150 rpm. They were subsequently centrifuged at 10,000 *g* for 6 min to separate the supernatant from the fungal biomass. The mycelium was dried at 60°C for 2 days and weighed using a precision balance (Mettler AE 163 analytical). One hundred μL of the supernatant was mixed with 400 μL of Salkowski’s reagent, which is a solution comprising 0.5 M FeCl_3_ and 35% perchloric acid (Platt and Thimann, 1956). The production of IAA was evidenced by the development of a pink color after a 30 min incubation in the dark. A semi-quantitative measure of the quantity of produced IAA was obtained by measuring the absorbance of the solutions supplemented with tryptophan at a wavelength of 530 nm, subtracting the absorbance of the solution without tryptophan and comparing it to a standard curve of IAA. The semi-quantitative concentrations of IAA were expressed per gram of mycelium (μg/g).

### Zinc and Cadmium Mobilization From the Soil

The ability of the seven fungal strains to mobilize Cd and Zn from the soil was estimated using the fungal extracts from the previous culture made in PDB without tryptophan (*n* = 3). The supernatants were filtered at 0.2 μm, and the pH of the filtrates was measured. Four milliliters of the filtrates were rotary shaken for 2 h at room temperature with 0.8 g of sterilized and dried Cd/Zn-contaminated soil used for the inoculation assay. Controls consisted of 0.8 g aliquots of the same soil incubated with 4 ml of fresh PDB with the pH adjusted to 3.4, 3.9, or 4.2 (pH range of the fungal extracts) or without adjustment (pH 5.2, corresponding to the original pH of the medium used for the fungal cultures). After centrifugation (7,000 rpm, 5 min), filtration at 0.45 μm and acidification at 5% with HNO_3_, the concentrations of Zn and Cd in the filtrates were determined by inductively coupled plasma optical emission spectrometry (ICP-OES, iCap 600, Thermo Fischer Scientific, Pittsburgh, PA, United States). Cadmium and Zn concentrations were expressed per g of mycelium. The ability to mobilize Zn and Cd from contaminated soil was estimated by measuring the difference in Cd and Zn concentrations (μg/g of soil per g of mycelium) between soil mixed with fungal extract and soil mixed with pH 5.2 PDB.

### Phosphate and Zinc Solubilization Activity

The ability of the seven fungal strains to solubilize phosphate and Zn was determined by inoculating the strains onto solid Pachlewski medium [2.3 g/L C_4_H_12_N_2_O_6,_ 0.5 g/l KCl, 1 g/L MgSO_4_, 7H_2_O, 5 g/L maltose, 20 g/L glucose, 10 μL/L thiamine HCl (1 g/L) and 100 μL/L Kanieltra solution, pH 5.5] supplemented with 12.5 g/L Ca_3_(PO_4_)_2_, 3 g/L ZnO, 5.5 g/L ZnCO_3_ or 5.25 g/L Zn_3_(PO_4_)_2_ (*n* = 4). Agar plates were covered with a sterile cellophane membrane and inoculated with 1 cm^2^ agar mycelial plugs. After 7 days of culture at 24°C, the diameters of both the colonies and the solubilization halos were measured. Finally, the solubilization index was calculated as the “diameter of the solubilization halo” over the “diameter of the colony” ratio.

### Siderophore Production

The siderophore production of the seven fungal strains was tested on chrome azurol S agar plates (*n* = 4). Fungal strains were grown on plates half-filled with Fe-free M9 medium (3 g/L C_4_H_12_N_2_O_6,_ 1 g/L NH_4_Cl, 6 g/L Na_2_HPO_4_, 0.5 g/L NaCl, and 4 g/L glucose). After 12 days of growth at 24°C, fungal colonies were removed together with the cellophane membranes, and the plates were filled with a CAS-blue agar overlay. The overlay medium (pH 6.8) was made of (per L) chrome azurol S (CAS) 60.5 mg, hexadecyltrimetyl ammonium bromide (HDTMA) 72.9 mg, piperazine-1,4-bis(2-ethanesulfonic acid) (PIPES) 30.24 g, 20 mL of FeCl_3_ (10 mM) prepared in HCl (100 mM) and agarose (0.9%, w/v) (Pérez-Miranda et al., 2007). A change in color from blue to orange observed in the overlaid medium evidenced the production of siderophores. The siderophore index was calculated as the “diameter of the orange halo” over the “diameter of the colony” ratio.

### Experimental Design of the Pot Experiment

A pot experiment was conducted with sandy-loamy soil taken from the top horizon of agricultural soil located at Chenevières (Grand Est, France), which was previously spiked with Cd, Pb, Cu, and Zn and used for the cultivation of *N. caerulescens* as part of previous studies (Sterckeman et al., 2017, 2019). At the beginning of the present study, the Cd and Zn concentrations were 3.6 and 649 mg/kg, respectively. Some physico-chemical parameters of the soil are provided in Supplementary Table 1. The seeds of *N. caerulescens* originated from a metallicolous population from a mining site located in Ganges [Occitanie, France, Gonneau et al. (2014)]. The seeds were sown in a sterilized mix (v/v) of compost (75%) and sand (25%) and placed in a growth chamber. Seven weeks after germination, plants of similar developmental stages were selected for the pot experiment.

In the present study, we tested seven fungal endophytic strains (DBF60, DBF79, DBF81, DBF107, DBF108, DBF129, and DBF159) originating from the collection of strains isolated from *N. caerulescens*. They were selected based on their potential as PGP fungi, according to their description in the literature. The production of inoculum and the soil inoculation procedure are fully described in Yung et al. (2021b). As a control treatment (CTRL), we mixed one aliquot of soil with perlite containing fungus-free agar plugs. Five replicates were considered for each fungal treatment, and four were considered for the mock-inoculated treatment. Seedlings were transplanted into 40 plastic pots (8 treatments × 5 replicates) of 500 mL volume (8.6 cm in diameter) previously loaded with 400 g of the inoculated soils. The pots were then placed in a growth chamber with 16 h of light at 22°C and 8 h of darkness at 18°C, 70% relative humidity and a photon flux density of 200 μmol photons m^–2^ s^–1^ in the PAR range. Water was supplied every 2 or 3 days to 80% of the field capacity.

### Estimation of NBI and Pigment Indices

Chlorophyl, flavonoid and anthocyanin contents in leaves of *N. caerulescens* and the nitrogen balance index (NBI) were measured using a dual-excitation fluorimeter DUALEX^®^ SCIENTIFIC+ (Force-A, Orsay, France). Full details about the analytical method are available in Yung et al. (2021b). These measurements were carried out on five mature leaves per plant at the end of the experiment.

### Plant Harvesting, Biomass Determination and Analysis of Trace and Major Elements

After 8 weeks of growth, roots and shoots were harvested and separated. Shoots were thoroughly washed with tap water and rinsed with deionized water. Soil was removed from the roots first by washing them with tap water and then by immersing them in 37.6 mM tetrasodium diphosphate for 16 h. The plant samples were then dried at 70°C for 48 h and weighed. Dried samples (0.5 g) were ground and digested according to the protocol detailed in Yung et al. (2021b). Trace and major elements were analyzed by ICP-OES. Control samples from *N. caerulescens* with known compositions (according to internal analyses carried out by INRA-USRAVE, Villenave d’Ornon, France), as well as a certified solution (EU-H-2, SCP Science, Courtaboeuf, France), were included in all analyses as quality controls.

### Estimation of the Rate of Fungal Colonization of Plant Roots

To estimate the colonization rate of plant roots by the inoculated endophytes, root segments were labeled with 20 μg/mL WGA-AF^®^488 (Invitrogen, France) (Berthelot et al., 2016) and observed by fluorescence microscopy. Labeled roots were cut into 10 mm segments (20 per plant) that were assessed separately using a microscope. The rate of root colonization (semi-quantitative) by fungal strains was estimated by assessing the frequency of microsclerotia and typical intracellular hyphae.

### Statistical Analyses

Statistical analyses were performed with R software v.3.5.1 (R Development Core Team, 2015). All statistical tests were considered significant at *P* ≤ 0.05. The mean values of the variables obtained from the *in vitro* tests of the inoculated strains were compared using Tukey’s HSD or the Kruskal–Wallis *post hoc* test to classify the strains according to their PGP abilities and their capacity to modify the mobility of Cd and/or Zn in the soil.

A multiple-factor analysis (MFA, “FactomineR” package) was used to evaluate the influence of the seven tested strains on (i) plant shoot and root biomass production, (ii) leaf pigments and NBI, and (iii) the concentrations of trace and major elements in roots and shoots. The latter variables and the amounts of extracted Cd and Zn (concentration of Cd/Zn in leaves × biomass) were then analyzed by comparing the CTRL treatment with the seven fungal treatments using an analysis of variance (ANOVA) followed by a Dunnett test in the “multcomp” package. The adequacy of the data from each measurement with respect to the ANOVA models was verified by examining the residuals for independence (Durbin-Watson test, “car” package), normality (Shapiro test) and homogeneity (Levene test). If the latter assumptions were not validated after “sqrt” or “log” transformation of the data, a Wilcoxon pairwise test with a Holm correction of the *p*-value was used.

## Results

### Taxonomic and Functional Description of the Fungal Isolates

Among the collection of endophytic strains isolated from the parts of *N. caerulescens*, the seven strains studied in the present study were isolated from populations originating from two calamine stations (Ganges and Montdardier; Occitanie, France) and two non-metallicolous stations located in Croix des Moinats (Grand Est, France) and Baraquette (Occitanie, France) (Gonneau, 2014). DBF79, DBF81 and DBF129 were isolated from leaves, while DBF60, DBF107, DBF108 and DBF159 were isolated from roots. The molecular identification enabled assignment, with sequence identities of 99–100% (Table 1), of the following affiliations. DBF60 (*Metapochonia rubescens*), DBF81 (*Trichoderma harzianum*) and DBF159 (*Microdochium bolleyi*) belong to Sordariomycetes, DBF79 (*Alternaria thlaspis*), DBF107 (*Cladosporium* sp.), and DBF129 (*Cladosporium cladosporioides*) belong to Dothideomycetes, and DBF108 (*Phialophora mustea*) belongs to Eurotiomycetes.

**TABLE 1:** Taxonomic identification of the fungal isolates and the origin of their host plant.

Isolate code	Geographic origin	Taxonomic affiliation	GenBank accession number	Closest accession number	Sequence identity (%)
DBF107	Ganges (CAL)	*Cladosporium* sp.	MW750568	MH118272.1	100
DBF108	Ganges (CAL)	*Phialophora mustea*	MW750569	MT576440.1	100
DBF129	Baraquette (N.MET)	*Cladosporium cladosporioides*	MW750570	MK656446.1	99
DBF159	Montardier (CAL)	*Microdochium bolleyi*	MW750571	MH319854.1	100
DBF60	Croix des Moinats (N.MET)	*Metapochonia rubescens*	MW750565	AB709859.1	99
DBF79	Ganges (CAL)	*Alternaria thlaspis*	MW750566	JN383495.1	99
DBF81	Ganges (CAL)	*Trichoderma harzianum*	MW750567	MT584872.1	100

*CAL, calamine soil; N.MET, non-metallicolous soil.*

The isolates were further characterized for their ability to promote plant growth through *in vitro* tests. To determine the ability of the isolates to assist Fe uptake by plants, siderophore production using the CAS-blue agar assay was carried out. The color change of agar from blue to orange was only observed in the case of DBF60, indicating that this strain was the only one able to produce siderophores (Table 2). The ability of the tested strains to produce IAA was determined through a colorimetric assay. The seven isolates had the ability to produce IAA with concentrations ranging from 1.0 to 4.1 μg/g DW of mycelium (Table 2). DBF81 produced the lowest IAA quantities. Tricalcium phosphate and various forms of Zn supplemented in Pachlewski medium were used to test the ability of the isolates to solubilize insoluble phosphate and Zn. All isolates had the ability to solubilize tricalcium phosphate (Table 2). DBF60, DBF81, and DBF159 were also able to solubilize Zn_3_(PO_4_)_2_. Moreover, DBF60 and DBF81 had the ability to solubilize ZnCO_3_ and ZnO (Table 2).

**TABLE 2:** Plant growth promotion properties of the seven endophytic isolates.

Strain	Ca_3_(PO_4_)_2_	Zn_3_(PO_4_)_2_	ZnCO_3_	ZnO	Siderophore	IAA
	Solubilization^1^	Solubilization^1^	Solubilization^1^	Solubilization^1^	production^2^	production^3^ (μg/g DW mycelium)
DBF107	1.00 ± 0.04^a^	0^c^	0^b^	0^b^	0^b^	4.1 ± 0.5^a^
DBF108	0.87 ± 0.07^abc^	0^c^	0^b^	0^b^	0^b^	3.0 ± 1.7^ab^
DBF129	0.56 ± 0.68^abc^	0^c^	0^b^	0^b^	0^b^	1.7 ± 0.8^ab^
DBF159	0.55 ± 0.12^abc^	0.92 ± 0.42^ab^	0^b^	0^b^	0^b^	2.3 ± 0.9^ab^
DBF60	1.39 ± 0.70^ab^	1.10 ± 0.10^a^	0.32 ± 0.23^a^	0.44 ± 0.51^ab^	1.44 ± 0.66^a^	1.8 ± 0.4^ab^
DBF79	0.27 ± 0.19^c^	0^c^	0^b^	0^b^	0^b^	2.4 ± 0.4^ab^
DBF81	0.50 ± 0.12^bc^	0.54 ± 0.07^b^	0.38 ± 0.05^a^	0.70 ± 0.11^a^	0^b^	1.0 ± 0.8^b^

*^1^Phosphate and zinc solubilization by the endophytic strains were recorded on agar plates after 2 weeks of growth. The solubilization index was calculated as the “diameter of the solubilization halo” over the “diameter of the colony” ratio.*

*^2^Siderophore production by the endophytic strains was recorded on agar plates after 2 weeks of growth. The index was calculated as the “diameter of the orange halo revealing the presence of Fe/siderophore complexes” over the “diameter of the colony” ratio.*

*^3^IAA production (semi-quantitative) was measured in liquid growth medium after 2 weeks of culture in minimal medium supplemented with tryptophan. Values are the means of three (IAA) or four (other tests) replicates ± SE. Significant differences (*P* < 0.05, Kruskal–Wallis *post hoc* test) between strains are indicated by different letters.*

The ability of the isolates to mobilize metals was further tested by measuring the concentrations of Zn and Cd mobilized from contaminated soil by the filtrates of the fungal cultures. All strains had the ability to increase the mobility of Zn and Cd from the soil when compared to the control medium (pH 5.2; no fungus cultivated; *P* < 0.05). DBF81, DBF107, and DBF129 had the highest ability to mobilize both Zn and Cd (Table 3). Filtrates obtained from the culture of DBF81, DBF107, and DBF129 had pH values spanning from 3.3 to 3.6. The filtrates of the other strains were less acidic (pH ≥ 3.9) (Supplementary Figure 1). A highly significant negative correlation (*r* = −0.90; *P* < 0.001) between the pH and Cd concentrations mobilized by the fungal filtrates was found (Supplementary Figure 1). The same finding was observed for Zn (*r* = 0.91; *P* < 0.001; Supplementary Figure 1). However, according to the slopes of regression lines drawn for fungal extracts and for control media (Supplementary Figure 1), fungal extracts, particularly the DBF60, DBF81, and DBF129 extracts, mobilized more Cd and Zn compared to the control media adjusted to equivalent pH values.

**TABLE 3:** Ability of the seven endophytic isolates to mobilize Zn and Cd from contaminated soil.

Fungal extract	Cd mobilized from soil		Zn mobilized from soil	
	μg/g soil/g biomass	Increase (%)	μg/g soil/g biomass	Increase (%)
DBF107	0.420.32^ab^	+417	67.160.8^ab^	+500
DBF108	0.110.15^b^	+690	18.015.7^b^	+949
DBF129	0.550.10^ab^	+680	92.818.2^ab^	+984
DBF159	0.190.13^b^	+431	25.919.6^b^	+576
DBF60	0.240.13^b^	+200	33.317.8^b^	+269
DBF79	0.050.1^b^	+160	8.31.3^b^	+171
DBF81	0.800.08^a^	+275	126.89.6^a^	+309

*The means ± SD (*n* = 3) of Cd and Zn concentrations in the soil extracts are presented in μg/g soil DW/g biomass DW. Different letters represent significant differences between strains (Kruskal–Wallis *post hoc* test). For each strain, the increase (%) in Cd and Zn concentrations extracted from the soil was calculated as follows: 100 × [TE]_*fungal medium*_/[TE]_*control medium at pH 5*__.2_.*

### Ability of the Endophytic Isolates to Colonize the Roots of *Noccaea caerulescens*

**FIGURE 1:**
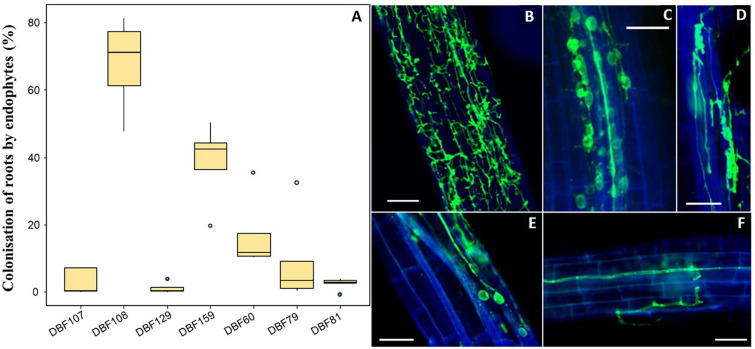
Root colonization of *N. caerulescens* inoculated by the endophytic isolates. **(A)** Level of fungal colonization for each strain (*n* = 5) based on the fluorescence microscopy observation of fungal structures within roots after labeling with WGA-AF488. Fungal structures were absent in mock-inoculated plants. **(B)** Intracellular colonization of the entire roots by DBF60. **(C)** Intracellular hyphae associated with conidiospores in DBF107-inoculated plants. **(D)** Contracted hyphae forming microsclerotia in DBF108-inoculated plants. **(E)** Intracellular hyphae associated with conidiospores in DBF60-inoculated plants. **(F)** Intracellular hyphae parallel to the vascular tissues and following the wall of cortical cells. Scales bars = 50 μm.

**FIGURE 2:**
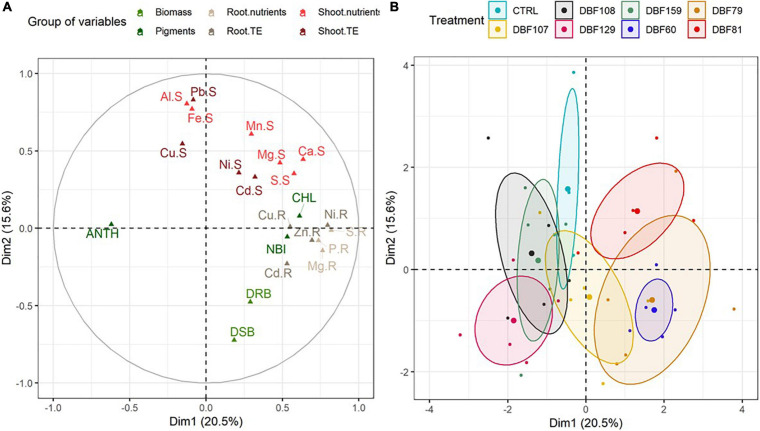
Multiple factor analysis included plant biomass [dry shoot biomass (DSB) and dry root biomass (DRB)], concentration of trace and major elements in roots (“.R”) and shoots (“.S”), and pigment index [anthocyanin (“ANT”), chlorophyll (“CHL”) and flavonoid (“FLA”)] variables for *N. caerulescens* grown in metal-contaminated soil and inoculated with seven endophytes. **(A)** Projections of element vectors on the two dimensions. **(B)** Projection of the groups [plants inoculated with the fungal strains and mock-inoculated (CTRL)], with confidence ellipses.

After inoculation with the seven DSE strains (DBF60, DBF79, DBF81, DBF107, DBF108, DBF129, and DBF159), typical structures of fungal endophytes in labeled roots were observed and quantified. We detected thin extracellular hyphae located on the root surface and within root tissues for all endophyte-inoculated treatments. Notably, twisting septate hyphae located within the cortical cells of roots were also found (Figure 1D). The root surface of DBF60-inoculated plants was highly colonized by fungal hyphae (Figure 1B), although intracellular hyphae associated with conidiospores were also detected (Figure 1E). In the case of DBF79 and DBF107, hyphae were often parallel to the vascular tissues (Figure 1F). For most of the inoculated roots and particularly for DBF79- and DBF129-inoculated plants, we detected a high number of intracellular conidiospores (Figure 2C). Contracted hyphae forming intracellular structures such as microsclerotia were observed in DBF108-inoculated roots (Figure 1D). However, the success of root colonization by the endophytes depended on the strain (Figure 1A). With a mean colonization rate <5%, DBF81, DBF107, and DBF129 poorly developed within the roots of *N. caerulescens*. DBF79- and DBF60-inoculated plants had mean colonization rates of 9.3 and 17.0%, respectively, corresponding to intermediate values. Conversely, DBF159 and DBF108 highly colonized the roots of *N. caerulescens*, with mean colonization rates of 38.6 and 67.8%, respectively.

### Influence of Fungal Inoculation on the Biomass of *Noccaea caerulescens*

**FIGURE 3:**
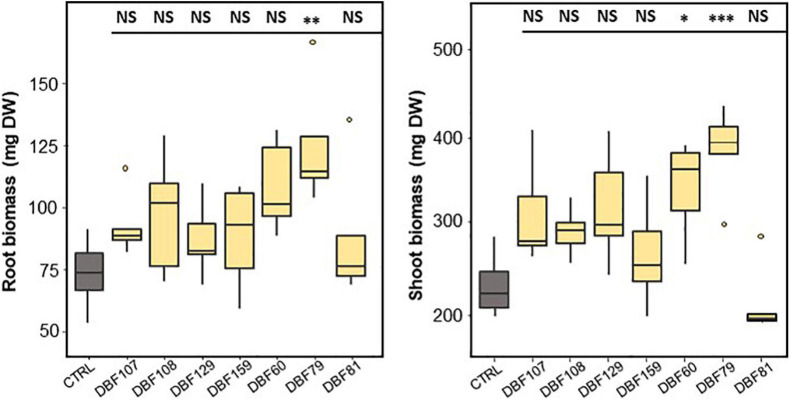
Effect of endophyte inoculation on the root (left) and shoot (right) biomass production of *N. caerulescens*. Plants were grown for 2 months on metal-contaminated soil and were either inoculated with a fungal strain (*n* = 5) or mock-inoculated (CTRL, *n* = 4). Significant differences (ANOVA, Dunnett test) between the CTRL condition and the fungal treatments are represented with the following legend: ^∗^*P* < 0.05; ^∗∗^*P* < 0.01; ^∗∗∗^*P* < 0.001.

As suggested by the MFA, the contribution of variables related to growth parameters [dry shoot biomass (DSB) and dry root biomass (DRB)] to the variability was quite high (Figure 2A). At the end of cultivation, the mean biomass of mock-inoculated *N. caerulescens* was 61 mg DW for roots and 206 mg DW for shoots, while endophyte-inoculated treatments ranged from 87 to 124 mg DW for roots and from 215 to 385 mg DW for shoots (Figure 3).

DBF79-inoculated plants showed a significant effect on biomass production by increasing the mean root (*P* < 0.01) and shoot biomass (*P* < 0.001) by 69 and 65%, respectively, compared to those of the mock-inoculated plants (Figure 3). DBF60 also increased the biomass production of *N. caerulescens* by 48% for roots (not significant) and 47% for shoots (*P* < 0.05) compared to the CTRL treatment. Plants inoculated with DBF107, DBF108, DBF129, and DBF159 tended to produce more root and shoot biomass; however, the substantial inter-sample variability did not allow us to confirm this result statistically (Figure 3). This tendency was not found for plants inoculated with DBF81.

### Influence of Endophytes on the Leaf Pigments and Mineral Nutrition of *Noccaea caerulescens*

**FIGURE 4:**
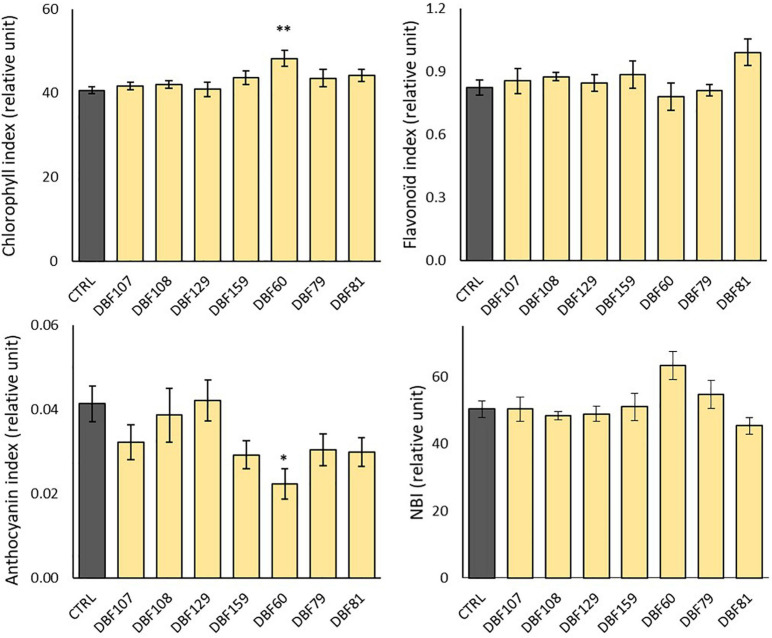
Effect of endophyte inoculation on the leaf pigment contents and nitrogen balance index (NBI) of *N. caerulescens*. Plants were grown for 2 months on metal-contaminated soil and were either inoculated with a fungal endophyte strain or mock-inoculated (CTRL). Data are the means ± SE (*n* = 5 for the fungal treatments and *n* = 4 for the mock-inoculated treatment). Significant differences (ANOVA, Dunnett or Wilcoxon pairwise test) between the CTRL condition and the fungal treatments are represented with the following legend: ^∗^*P* < 0.05; ^∗∗^*P* < 0.01.

The chlorophyll content in the leaves of DBF60-inoculated plants was significantly increased compared to that in the mock-inoculated plants (*P* < 0.01), while the anthocyanin content was reduced (*P* < 0.05) (Figure 4). The other endophytic strains had no significant influence on the content of leaf pigments or NBI (Figure 4). However, when compared to the CTRL treatment, inoculation with the six other strains tended to slightly decrease the anthocyanin index of leaves, while chlorophyll, flavonoid and NBI indices did not show any particular trend (Figure 4).

**FIGURE 5:**
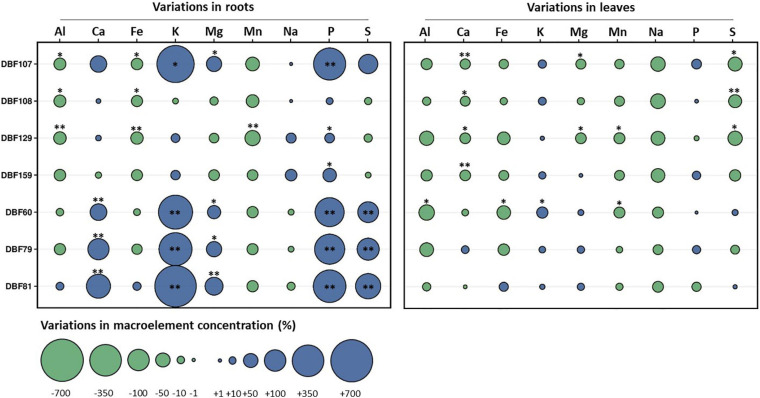
Effect of fungal endophytes on the elemental composition of roots and leaves of *N. caerulescens*. Data represent the variations (%) of the concentrations of macroelements in roots or leaves of endophyte-inoculated plants in comparison to those of the mock-inoculated plants. Asterisks denote significant differences (ANOVA, Dunnett test) between a given fungal treatment and the control condition with the following legend: ^∗∗^*P* < 0.01; ^∗^*P* < 0.05.

All strains influenced the mineral nutrition of *N. caerulescens* when inoculated, as they induced significant effects on the elemental composition of roots and shoots, although the effects were more pronounced in roots (Supplementary Table 2). According to the MFA, the groups of variables related to the root nutrient and TE status and shoot pigment content had the highest contributions to the variability for dimension one. Dimension two was mostly explained by the groups of variables related to the shoot nutrients and TE status and biomass production (Figure 2A and Supplementary Figure 2). The cluster corresponding to plants inoculated with DBF60, DBF79, DBF81, and DBF107 differed from those of the other treatments due to its different nutritional status (Figure 2B). The inoculation of *N. caerulescens* with DBF60, DBF79, and DBF81 increased the root concentrations of Ca (+86, +149, and +199%, respectively; *P* < 0.01), K (+401, +429, and +656%, respectively, *P* < 0.01), Mg (+48, +69, and +102%, respectively, *P* < 0.05), P (+302, +329, and +391%, respectively, *P* < 0.01) and S (+143, +164, and +223%, respectively, *P* < 0.01) compared to the CTRL (Figure 5). Among these three strains, only DBF60 had a significant influence on the elemental composition of leaves by increasing K concentrations by 32% and slightly decreasing Al,

Fe and Mn concentrations (*P* < 0.05) (Figure 5). DBF107 had a significant influence on the root concentrations of K (+518%, *P* < 0.05), Mg (+68%, *P* < 0.05), and P (+378%, *P* < 0.01) and on the leaf concentrations of Ca (−25%, *P* < 0.01), Mg (−23%, *P* < 0.05), and S (−55%, *P* < 0.05) (Figure 5). Inoculation of *N. caerulescens* with DBF108, DBF129, and DBF159 had only slight effects on the nutrient concentrations in leaves and roots (Figure 5).

### Influence of Fungal Strains on the Phytoextraction Potential of *Noccaea caerulescens*

Globally, the mean Cd concentrations in *N. caerulescens* were 22.3 mg/kg DW for roots and 23.3 mg/kg DW for leaves, which corresponds to values sixfold higher than the concentrations found in the soil. The mean Zn concentration of *N. caerulescens* roots was approximately twofold lower than the mean soil concentration, while leaves accumulated 1,662.7 mg Zn/kg DW, corresponding to 2.6-fold the mean soil concentration.

**FIGURE 6:**
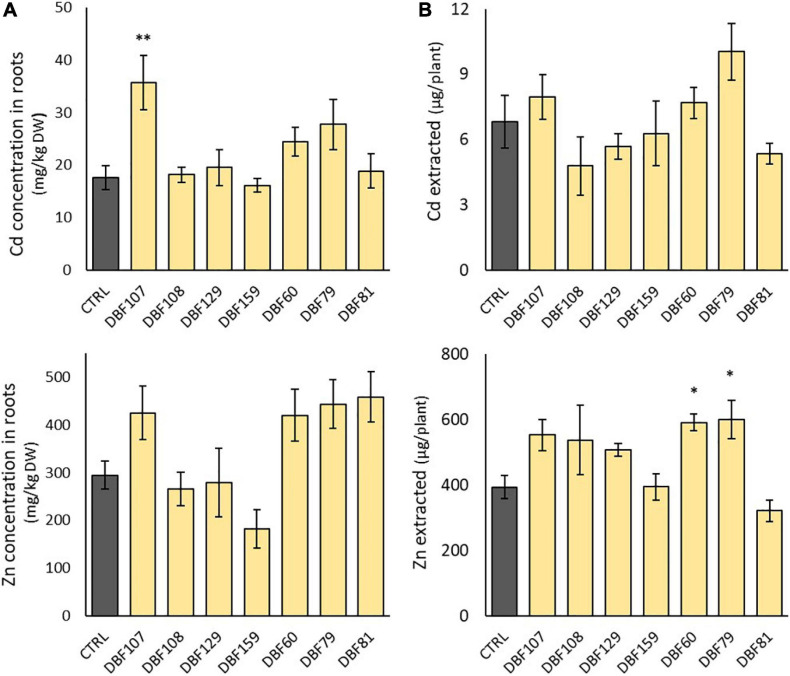
Effect of endophytes on Cd and Zn concentrations in the roots of *N. caerulescens* **(A)** and on the amount of Cd and Zn extracted by leaves of *N. caerulescens* **(B)**. Plants were grown for 2 months on metal-contaminated soil and were either inoculated with an endophytic strain or mock-inoculated (CTRL). Data are the means ± SE (*n* = 5 for the fungal treatments and *n* = 4 for the mock-inoculated treatment). Significant differences (ANOVA, Dunnett test) between the CTRL condition and the fungal treatments are represented with the following legend: ^∗^*P* < 0.05; ^∗∗^*P* < 0.01.

As previously observed for macroelements, the group comprising DBF107, DBF60, DBF79 and DBF81 tended to have higher concentrations of Cd and Zn in roots of *N. caerulescens* when inoculated, while DBF108, DBF129, and DBF159 showed no effect (Figure 6A). The concentrations of Cd in roots increased by 102% for DBF107-inoculated plants (*P* < 0.01), by 38% for DBF60-inoculated plants (not significant) and by 57% for DBF79-inoculated plants (not significant) compared to the mock-inoculated plants (Figure 6A). The latter endophytic strains as well as DBF81 also tended to increase Zn concentrations in roots from 43 to 56% compared with the CTRL treatment, although not significantly (Figure 6A). None of the tested strains significantly increased the TE concentrations in the leaves of *N. caerulescens* (Supplementary Table 3).

However, when focusing on the amount of Zn extracted from the soil by each plant (leaf biomass × Zn concentrations in leaves), DBF60- and DBF79-inoculated plants extracted +50 and +53% of Zn from the soil, respectively, compared with the mock-inoculated plants (*P* < 0.05) (Figure 6B). With the amount of Cd extracted by inoculated plants ranging from 4.8 to 10.0 μg/plant vs. 6.8 μg/plant for mock-inoculated plants, we did not detect any significant influence of the strains on the potential of *N. caerulescens* for Cd phytoextraction (Figure 6B).

## Discussion

### *Noccaea caerulescens* Hosts Several Taxa of Recognized PGP Fungal Endophytes

*Noccaea caerulescens* has been recently shown to be a potential candidate for fungal-assisted phytoextraction, as plant responses are beneficial when associated with certain host (isolated from the same species) (Ważny et al., 2021) and non-host (Yung et al., 2021b) endophytic strains under TE exposure. In the present study, we tested seven fungal endophytes isolated from non-metallicolous and calamine populations of *N. caerulescens* based on their taxonomy. Among the panel of isolated taxa, we selected seven taxa that have previously demonstrated promising direct PGP abilities (i.e., improvement of plant nutrition, growth, and/or stress alleviation) or indirect positive impacts by acting as biocontrol agents. We selected two strains belonging to *Cladosporium* sp. (DBF107) and *C. cladosporioides* (DBF129). Ważny et al. (2021) also isolated a strain of *C. cladosporioides* from the root of *N. caerulescens* that significantly increased plant biomass and stimulated the uptake and accumulation of Ni in both roots and shoots compared to non-inoculated controls. *Phialophora mustea* (DBF108) is a DSE with recognized PGP abilities, including the improvement of metal tolerance for the plant (Berthelot et al., 2017; Zhu et al., 2018). A strain of *P. mustea* isolated from poplar roots has already been proposed as a promising candidate to enhance the phytoextraction potential of *N. caerulescens* (Yung et al., 2021b). *Alternaria thlaspis* (syn: *Embellisia thlaspis;* DBF79) was described after isolation from the roots of *N. caerulescens* growing in soil with high levels of Zn and Pb (David et al., 2000). This species was also recently isolated from roots and leaves of calamine and serpentine *N. caerulescens* and tested for its PGP abilities on *N. caerulescens* (Ważny et al., 2021). *Microdochium bolleyi* (syn: *Idriella bolleyi*; DBF159) is a well-known species of DSE that has been extensively isolated from roots and shoots of several cereals and grasses (Gadd, 1981; David et al., 2016; Rothen et al., 2018). Although, it is sometimes considered as a pathogen, it is also considered non-pathogenic in some cases (Kirk and Deacon, 1987), with potential implications for organic matter mineralization, plant nutrition (Mandyam et al., 2010), and biocontrol agents (Jadubansa et al., 1994). Indeed, Shadmani et al. (2021) recently demonstrated that *M. Bolleyi* could be exploited to improve the potential of barley for the remediation of Cd-contaminated sites. *Trichoderma harzanium* (DBF81) is a well-studied fungus used as a biocontrol agent in agriculture against plant pathogens and nematodes (Lorito et al., 2010). Some strains are able to induce plant defenses (Yedidia et al., 1999) and stimulate plant growth and development (Yedidia et al., 2001; Hermosa et al., 2012) by establishing a molecular dialog with the roots (Mendoza-Mendoza et al., 2018). These beneficial effects of T. *harzanium* have been mainly demonstrated on horticultural plants. Recently, Poveda et al. (2019) showed that *T. harzianum* favors the access of arbuscular mycorrhizal fungi to non-host Brassicaceae roots, providing new perspectives for agronomy. *Metapochonia rubescens* was mainly studied for its application as a biocontrol agent as a facultative parasite of major plant-parasitic nematodes (Moosavi and Zare, 2020).

### Fungal Endophytes Differentially Colonized the Roots of *Noccaea caerulescens*

While the method used for estimating the rate of fungal colonization is semi-quantitative, it enabled to monitor the success of the inoculations, by confirming the presence of fungi associated with plant roots and providing an estimation of the extent to which the colonization occurred (Ayliffe et al., 2013). Although all of the selected strains here were indigenous and belonged to taxa that have been described as potential auxiliary endophytic fungi in other contexts, under metallic stress conditions, the success of their association (i.e., ability to colonize roots and positive host plant response) with *N. caerulescens* was variable. Although DBF79, DBF81, and DBF129 were isolated from the leaves of *N. caerulescens* in the present study, these taxa are not strict phyllospheric fungi. When reinoculated with *N. caerulescens* by mixing the inocula with the soil, these strains indeed colonized the plant roots, although with a low rate of colonization. According to the literature, *A. thlaspi* (DBF79) and *C. cladosporioides* (DBF129) have already been isolated from *N. caerulescens* roots (David et al., 2000; Ważny et al., 2021), whereas *T. harzanium* (DBF81) is a well-known soil-borne filamentous fungus (Lorito et al., 2010), suggesting its probable presence in the rhizosphere. Fungal structural characteristics of endophytic fungi, including twisting hyphae, have also been observed for all of the other strains.

### Three Endophytic Strains Had a Neutral Effect on *Noccaea caerulescens*

An endophytic relationship, which can vary from parasitism to mutualism (Freeman and Rodriguez, 1993; Mandyam and Jumpponen, 2005; Newsham, 2011; Mayerhofer et al., 2013), has been demonstrated to be context-dependent, with determining factors such as environmental conditions (Álvarez-Loayza et al., 2011), the composition of the plant-associated microbial community (Junker et al., 2012) and the plant genotype (Wheeler et al., 2019). Long-term endophyte-host interactions should be considered a basis for mutual interactions, with both sides constantly shaping the organization and structure of the other, resulting in a beneficial relation (Saikkonen et al., 2004). This plant-microbiome superorganism, which is also called a holobiont, is subjected to evolutionary forces that may result in the coevolution of host symbiont interactions (Rosenberg and Zilber-Rosenberg, 2018), where microorganisms could be considered a genetic extension of the host plant genome, favoring hologenome plasticity and thus plant adaptability to its environment (Durand et al., 2021). The success of inoculation could be mainly dependent on the host-symbiont combination at the species level (Fernando and Currah, 1996; Newsham, 2011; Mayerhofer et al., 2013; Berthelot et al., 2016), but in some cases, the plant population-fungal strain level appeared to be important (Ważny et al., 2021).

In the present study, the effects of reinoculating *N. caerulescens* with their associated endophytes were neutral to beneficial, depending on the strain. The lack of effect of DBF129 could be related to its low ability to colonize the roots of inoculated plants. The fact that DBF159 and DBF108 were highly abundant within the cortical cells of *N. caerulescens* roots and that they have been identified as potential PGP taxa elsewhere (Mandyam et al., 2010; Yung et al., 2021b), while having no effect on plant growth and/or nutrition in the present study, confirms the somewhat variable outcomes of host-endophyte interactions. Interestingly, we showed in our previous study that the non-host strain of *P. mustea* (Pr27, isolated from poplar roots) had a positive effect on the mineral nutrition of *N. caerulescens* (Yung et al., 2021b), while the indigenous strain DBF108 belonging to the same species had no effect in the present study. These results do not support assumptions in favor of the necessity of long-term adaptation of the plant and fungal endophytes resulting in mutualism.

### Four Endophytic Strains Highly Enhanced the Mineral Nutrition of *Noccaea caerulescens*

The most pronounced effects of the isolates, particularly those of DBF60, DBF79, DBF81, and DBF107, concerned the root elemental concentrations of Ca, Mg, K, P, and S, with a fivefold increase in some cases. Our study indicates a good potential of the DBF60 and DBF81 strains to solubilize inorganically bound phosphate, which is consistent with the improvement of the P concentration in plant roots. *T. harzanium* (DBF81) is known to be involved in mineral solubilization (Altomare et al., 1999; Rawat and Tewari, 2011; Tallapragada and Gudimi, 2011; Bader et al., 2020) through various mechanisms, including solubilization *via* acidification, redox reactions, chelation and hydrolysis (Li et al., 2015). According to several authors, the main mechanism by which fungal endophytes solubilize phosphate and other minerals is the production of organic acids that lowers the pH of the soil, consequently causing the solubilization of insoluble minerals (Chhabra and Dowling, 2017; Adhikari and Pandey, 2019; Mehta et al., 2019). All of the tested fungal strains, except DBF79, reduced the pH of their media. Therefore, improvement of the mineral nutrition of DBF79-inoculated plants is unlikely due to the sole mechanism of acidification. The rhizophagy, in which fungi can be the prey of roots and serve as nourishing factors to improve plant nutrition and growth, would be a possible hypothesis explaining the improvement of the root elemental composition of DBF79-inoculated plants in the absence of strain-mediated acidification (Paungfoo-Lonhienne et al., 2010; White et al., 2018). However, we cannot rule out an important acidification of DBF79 in the rhizosphere. To examine this hypothesis, further experiments are needed to locally measure the pH in the rhizosphere of both non-inoculated and inoculated plants.

### DBF60 and DBF79 Are Two Promising Fungal Isolates for Improving the Zn Phytoextraction Potential of *Noccaea caerulescens*

In the case of DBF60- and DBF79-inoculated plants, the improvement of mineral nutrition was concomitant with an increase in plant biomass production of the same order of magnitude as those detected for similar inoculation assays using plants exposed to TE (Khan et al., 2017; Rozpądek et al., 2018; Sharma et al., 2019; Ważny et al., 2021). According to the literature, such tripartite relationships (i.e., fungal colonization *vs.* nutrient content *vs.* plant biomass) have not been reported in many studies (Berthelot et al., 2019). Our results for *A. thlaspis* (DBF79) confirmed that this species could promote the growth of *N. caerulescens*, while this is the first report of direct PGP abilities of *M. rubescens* (DBF60). The comparable ability of the seven endophytes to produce IAA suggests that this phytohormone, which is well known to improve root development and enhance root exudation and mineral uptake (Fu et al., 2015), alone cannot explain the higher increase in biomass for DBF60- and DBF79-inoculated plants. However, the correlation between IAA synthesis and growth promotion in soil-based systems is scarce (Nieto-Jacobo et al., 2017) and deserves further attention in the future. In addition to the improvement of plant mineral nutrition, DBF60 also induced changes in the metabolism of *N. caerulescens* by increasing chlorophyll and decreasing anthocyanin contents, suggesting an enhancement of primary metabolism at the cost of secondary metabolism. The promotion of secondary metabolism through the production of anthocyanins is commonly observed when plants face biotic or abiotic stressors (Landi et al., 2015). In our context, the decrease in the anthocyanin concentration in favor of chlorophyll indicates that DBF60 could help plants alleviate metallic stress.

Due to their ability to increase the aboveground biomass of *N. caerulescens* and their tendency to increase Zn concentrations in leaves (statistically not significant), DBF60 and DBF79 enhanced the Zn phytoextraction potential (+ 50%) of *N. caerulescens*. This improvement was slightly better than that induced by the non-host Pr27 strain (amount of Zn extracted increased by 30%) (Yung et al., 2021b).

The mechanism leading to the increase in TE in a plant could be related to the capacity of endophytes to improve TE bioavailability through the release of metal chelating agents (e.g., siderophores, biosurfactants or organic acids), acidification of soils and redox activity (Deng and Cao, 2017; Domka et al., 2019). The results from our assay revealed that the quantity of Cd and Zn mobilized from the soil by the fungal extracts of DBF60 and DBF79 was the lowest. These results suggest that the ability of fungi to mobilize TE from the soil is not representative of the mechanisms occurring in the case of plant-fungus interactions. According to the results obtained from *in vitro* tests, the enhancement of plant nutrition and Zn phytoextraction mediated by DBF60 is partly associated with its ability to (i) solubilize various forms of insoluble Zn and P, (ii) acidify its environment, and (iii) produce siderophores. However, the effect of DBF79 on plant nutrition and Zn phytoextraction has not been corroborated by *in vitro* tests, confirming the necessity to adopt *in planta* approaches to better understand the solubilization level mediated by fungal endophytes.

The selected fungal endophytes were tested for their ability to improve the TE phytoextraction of *N. caerulescens* in sterilized soil, limiting competition with native microflora and consequently mitigating the risk of inoculation failure. It would be interesting to conduct a similar study comparing host and non-host PGP strains within the same system using non-sterilized soil to investigate the potential of these strains to tolerate competition with indigenous microflora.

## Conclusion

In conclusion, we demonstrated that *N. caerulescens* responses to fungal inoculation using native strains ranged from neutral to beneficial. Despite the seven tested strains had PGP abilities according to the *in vitro* tests, only two of them exhibited relevant effects to improve the phytoextraction potential of *N*. *caerulescens* when reinoculated. The improvement in plant elemental nutrition mediated by *M. rubescens* (DBF60) and *A. thlaspis* (DBF79) was significant and led to an increase in plant biomass and consequently to a higher amount of Zn extracted. These fungal endophytes could represent potential candidates for field applications using hyperaccumulating plants, with benefits for microbial-assisted phytoextraction and agromining.

## Data Availability Statement

The datasets presented in this study can be found in online repositories. The names of the repository/repositories and accession number(s) can be found in the article/Supplementary Material.

## Author Contributions

CS and DB designed the study. LY, CS, AA-B, and DB performed the material preparation, data collection, and analysis. LY, CS, and DB wrote the manuscript. All authors read and approved the final manuscript.

## Conflict of Interest

The authors declare that the research was conducted in the absence of any commercial or financial relationships that could be construed as a potential conflict of interest.
