# Supplementary material for: Natural Fungal Endophytes From Noccaea caerulescens Mediate Neutral to Positive Effects on Plant Biomass, Mineral Nutrition and Zn Phytoextraction
Source: Front Microbiol. 2021 Jul 6;12:689367. doi: 10.3389/fmicb.2021.689367 (PMC8290495; doi:10.3389/fmicb.2021.689367)
Supplement: Supplementary file 1 [file Data_Sheet_1.PDF]

Supplementary Material

Table S1. Physico-chemical characteristics, concentrations of major (cobaltihexammine extraction, cmol+/kg) and trace (total concentrations, mg/kg) elements of the Cd-Zn contaminated soil.

pH	C_{org}	N_{org}	C/N	$CaCO_3$ tot	CEC	K^+	Ca^{2+}	Mg^{2+}	Na^+	Cd	Cu	Ni	Pb	Zn
	%	%		%	cmol+/kg					mg/kg				
6.45	1.0	0.1	9.3	0.1	6.1	0.2	8.4	0.4	0.5	3.6	28.4	120.0	60.2	649

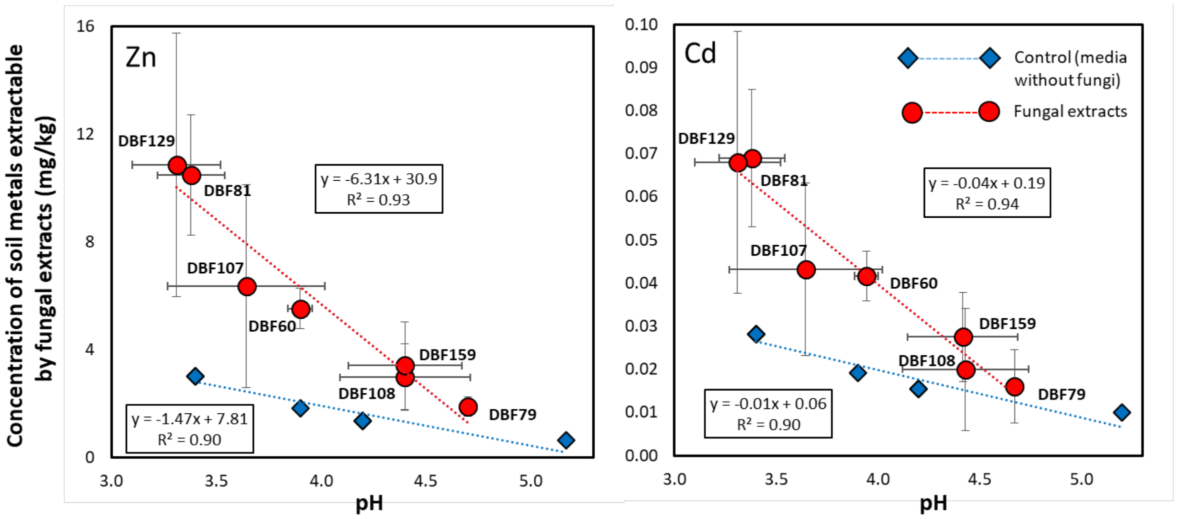

Figure S1. Relationship between the pH and the soil Cd and Zn concentration (mg/kg) extractable by the fungi (fungal strains cultured in PDB media) and control media (without fungi). Data are the means ± SD (n = 3 for the fungal treatments and n = 2 for the fungus-free PDB media).

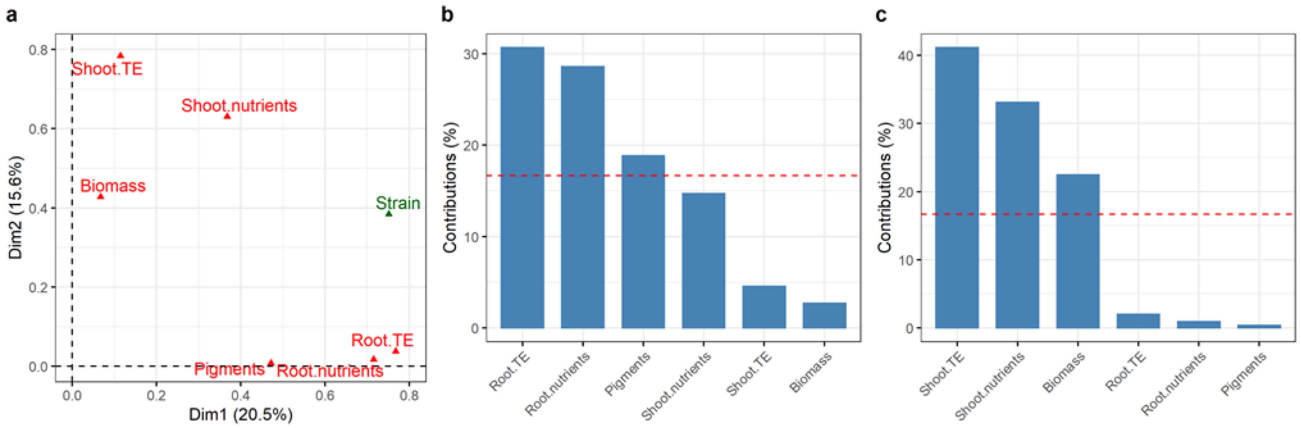

Figure S2. Multiple factor analysis included plant biomass [dry shoot biomass (DSB) and dry root biomass (DRB)], concentration of trace and major elements in roots (".R") and shoots (".S"), and pigment index [anthocyan ("ANT"), chlorophyll ("CHL") and flavonoid ("FLA")] variables for *N. caerulescens* grown in metal-contaminated soil and inoculated with seven fungal endophytes. (a) Projections of groups of variables in the two dimensions and their contributions to dimension 1 (b) and dimension 2 (c).

Table S2. Effect of endophyte inoculation on the elemental concentration (mg/kg DW) in roots and shoots of *N. caerulescens*. Plants were grown for two months on a trace metal-contaminated soil and where either inoculated with a fungal endophyte or mock-inoculated (CTRL). Data are the means ± SD (n = 5 for the fungal treatments and n = 4 for the mock-inoculated treatment). Significant differences (ANOVA, Dunnett test) between the CTRL condition and the fungal treatments are represented with the following legend: P < 0.05 (*); P < 0.01 (**); P < 0.001 (***).

**Elemental concentration in roots**									
	Al	Fe	Ca	K	Na	Mg	Mn	P	F
CTRL	576 ± 138	777 ± 69	525 ± 125	1025 ± 219	411 ± 96	324 ± 166	7068 ± 807	771 ± 36	1379 ± 122
DBF107	395 ±101 *	366 ±94 *	1437 ±488	5560 ±2831 *	7030 ±1137	654 ±154 *	212 ±72	3819 ±1301 **	3357 ±1156
DBF108	391 ±96 *	383 ±104 *	798 ±62	863 ±147	7037 ±949	335 ±80	255 ±20	859 ±78	1388 ±63
DBF129	364 ±28 **	329 ±31 **	813 ±106	1048 ±355	8693 ±1623	307 ±140	145 ±27 **	967 ±85 *	1317 ±94
DBF159	415 ±77	387 ±68	742 ±155	1077 ±397	9476 ±1682	279 ±131	328 ±56	1207 ±306 *	1464 ±128
DBF60	565 ±144	514 ±131	1454 ±295 **	4756 ±1847 **	6713 ±799	577 ±79 *	309 ±29	3210 ±622 **	3710 ±572 **
DBF79	428 ±151	414 ±125	1954 ±1666 **	4505 ±765 **	6731 ±1715	658 ±222 *	315 ±34	3424 ±708 **	4025 ±1042 **
DBF81	707 ±332	630 ±284	2341 ±1731 **	6795 ±242 **	6140 ±1407	785 ±158 **	309 ±50	3921 ±398 **	4924 ±840 **
**Elemental concentration in shoots**									
	Al	Fe	Ca	K	Na	Mg	Mn	P	S
CTRL	180 ± 157	16065 ± 1209	231 ± 151	25936 ± 2598	1460 ± 121	1138 ± 176	1668 ± 1065	3740 ± 774	9111 ± 2591
DBF107	123 ±73	183 ±74	12495 ±1495 **	25551 ±2584	1034 ±396	1157 ±196 *	924 ±117	4228 ±1433	3638 ±887 *
DBF108	153 ±128	207 ±117	12904 ±2764 *	25105 ±1841	959 ±294	1377 ±294	881 ±179	3481 ±719	4389 ±382 **
DBF129	74 ±40	128 ±50	11976 ±2255 *	22245 ±3822	1199 ±860	1084 ±44 *	805 ±157 *	3383 ±799	3371 ±705 *
DBF159	121 ±65	176 ±71	11974 ±2505 **	24497 ±4027	1237 ±575	1519 ±514	886 ±253	3969 ±922	5495 ±1307
DBF60	52 ±23 *	110 ±16 *	15429 ±1930	29617 ±2946 *	1821 ±1023	1581 ±147	804 ±130 *	3467 ±538	8563 ±2636
DBF79	83 ±66	151 ±78	18448 ±4232	23960 ±3291	2025 ±571	1654 ±386	1076 ±231	3934 ±1554	6777 ±2412
DBF81	153 ±63	270 ±97	16496 ±5286	23111 ±6844	1855 ±1610	1648 ±709	1044 ±270	2808 ±580	8238 ±2224

Table S3. Effect of endophyte inoculation on the concentrations (mg/kg DW) of Cd and Zn in shoots of *N. caerulescens*. Plants were grown for two months on a trace metal-contaminated soil and where either inoculated with a fungal endophyte or mock-inoculated (CTRL). Data are the means ± SD (n = 5 for the fungal treatments and n = 4 for the mock-inoculated treatment). Comparisons of means (ANOVA, Dunnett test) between the CTRL condition and the fungal treatments revealed no significant difference.

Treatment	Cd (mg/kg DW)	Zn (mg/kg DW)
DBF107	25.6 ± 6.5	1809 ± 486
DBF108	15.8 ± 7.8	1796 ± 615
DBF129	18.0 ± 3.8	1618 ± 244
DBF159	23.2 ± 9.2	1502 ± 340
DBF60	34.5 ± 27.7	1772 ± 396
DBF79	26.9 ± 10.7	1594 ± 449
DBF81	25.2 ± 5.3	1523 ± 429
